# Morphologic change of nerve and symptom relief are similar after mini-incision and endoscopic carpal tunnel release: a randomized trial

**DOI:** 10.1186/s12891-017-1438-z

**Published:** 2017-02-03

**Authors:** Won-Taek Oh, Ho-Jung Kang, Il-Hyun Koh, Jin-Young Jang, Yun-Rak Choi

**Affiliations:** Department of Orthopedic Surgery, Severance Hospital, Yonsei University College of Medicine, 50 Yonseiro, Seoul, Seodaemungu South Korea

**Keywords:** Carpal tunnel syndrome, Mini-incision, Endoscopic carpal tunnel release, Ultrasonography

## Abstract

**Background:**

The symptoms in carpal tunnel syndrome (CTS) can be ameliorated by open and endoscopic release of the transverse carpal ligament. It is unknown whether a mini-incision or endoscopic carpal tunnel release more effectively reverses the pathological changes that are observed in the median nerve in patients with CTS and these morphologic changes correlates with the subjective outcomes after carpal tunnel release. We hypothesized that (1) at 24 weeks after surgery, the subjective outcomes of mini-incision release and endoscopic release would not differ in patients with CTS; and (2) the ultrasonographic (US) morphology of the median nerve reverses similarly after mini-incision and endoscopic release; (3) the subjective outcomes correlates with these morphologic changes.

**Methods:**

Between November 2011 and January 2013, 67 patients with CTS in their dominant wrist were randomized to either mini-incision (*n* = 32) or endoscopic (*n* = 35) release. Each patient was assessed by both the Boston Carpal Tunnel Questionnaire (BCTQ) and the Disabilities of the Arm, Shoulder, and Hand (DASH) pre-operatively and 24 weeks’ post-operation. An US examination was conducted at both time points to measure the cross-sectional area (CSA) at the inlet, middle, and outlet (CSA-I, CSA-M and CSA-O) and the flattening ratio (FR) at the middle and outlet (FR-M and FR-O) of the median nerve.

**Results:**

The post-operative mean BCTQ and DASH scores were improved significantly from the pre-operative scores in both groups (*p* < 0.001). The mean CSA-I decreased and CSA-M and CSA-O increased similarly in both groups (by 3.3, 3.0, and 3.8 mm^2^ in the mini-incision group and 2.9, 3.1. and 2.7 mm^2^ in the endoscopic group. The mean FR-M/FR-O decreased similarly from 3.6/4.2 to 3.2/3.0 in the mini-incision group and 3.8/4.3 to 3.2/2.9 in the endoscopic group. There were no significant differences in the subjective outcome scores or median nerve measures between the two groups. Improvement in the BCTQ-S only was significantly correlated with changes in the CSA at the inlet.

**Conclusions:**

Mini-incision and endoscopic release both similarly relieved subjective symptoms and functions along with the pathological changes in the median nerve morphology along the carpal tunnel in patients with idiopathic CTS. Symptom relief after surgical decompression seems to correlate with reduced nerve swelling at carpal inlet and reversed nerve flattening inside carpal tunnel.

**Trial registration:**

This study was retrospectively registered in “ClinicalTrials.gov” at Oct 18th, 2013, and the registration number was NCT01972165.

## Background

Although carpal tunnel syndrome (CTS) can be diagnosed from signs and symptoms, conformational tests should be obtained in patients that require surgery [14]. Traditionally, electrodiagnostic tests have been used to confirm CTS; however, these tests are expensive, time consuming, and uncomfortable for patients. In addition, electrodiagnostic tests have relatively high false negative rates (16 to 34%) in patients with clinically defined CTS. Ultrasonography (US) has emerged as an alternative diagnostic test for CTS that is as sensitive and specific as the electrodiagnostic tests, and is also more comfortable and cost-effective for patients [8]. An increase in the cross-sectional area (CSA) of the median nerve at the inlet of the carpal tunnel is the most reliable US measure for the diagnosis of CTS, and is correlated with the electrodiagnostic severity [7, 12, 13, 20, 21]. A flattening of the median nerve in the distal carpal tunnel is another distinctive feature of CTS that can be measured by US [17].

If non-operative treatments for CTS such as local steroid injections, splinting, oral steroids, and ultrasound therapy fail, then a complete division of the transverse carpal ligament should be considered as a treatment option [4, 6, 10, 23]. The open (i.e., standard or mini-incision) and endoscopic release of the transverse carpal ligament are the most common surgical techniques for the treatment of CTS. It has been reported that endoscopic release results in less pain in the early post-operative period as well as a quicker return to work and fewer wound complications, and is preferred by patients despite a higher risk of median nerve injury [11, 23, 25]. Mini-incision release is a less technically demanding technique and has a lower rate of complications and costs [9]. Early reports on the mini-incision technique suggest that mini-incision release is as effective as endoscopic release [22, 27]. Although each technique has advantages and disadvantages, the subjective outcomes as reported by the patients 3 months or more after surgery are similar [11, 22, 27]. A few studies have shown that the mini-incision release technique decreases the pathologic swelling of the median nerve at the inlet of the carpal tunnel and increases the flattening ratio (FR) of the median nerve in the carpal tunnel [17, 24, 26]. Likewise, several publications on endoscopic release have also reported decreased CSA and increased FR of the median nerve in US measurement post-operatively [2, 5]. However, few studies have compared pathological changes for an open technique to an endoscopic technique.

We therefore conducted the current study to compare the subjective outcomes and US-measured morphological changes in the median nerve in patients with CTS who received either mini-incision or endoscopic release. We hypothesized that (1) subjective outcomes, as assessed by both the Boston Carpal Tunnel Questionnaire (BCTQ) symptom/function scores and the DASH scores, would be similar 24 weeks after either mini-incision or endoscopic carpal tunnel release; (2) changes in the morphology of the median nerve at each level of the carpal tunnel, as measured under high-resolution US, would be similar 24 weeks after either mini-incision or endoscopic carpal tunnel release; and (3) morphological changes would be correlated with improvements in subjective outcomes 24 weeks after mini-incision or endoscopic carpal tunnel release.

## Methods

This study was approved by the Institutional Review Board (IRB No. 1-2011-0072). All patients provided written informed consent before participating in this study. Only patients with idiopathic CTS that were confirmed by electrodiagnostic tests were included in the study.

Between November 2011 and January 2013, 93 consecutive patients with idiopathic CTS who were scheduled for carpal tunnel release were enrolled in the study. Carpal tunnel release was recommended for patients in whom clinical symptoms of tingling, pain, or weakness did not improve after at least 3 months of treatment with a splint, medication, and/or corticosteroid injections. The exclusion criteria included: (1) a history of wrist-area fracture or dislocation; (2) previous carpal tunnel release; (3) associated cervical radiculopathy, cubital tunnel syndrome, thoracic outlet syndrome, diabetes mellitus, hypothyroidism, arthritis, or Burger's disease; (4) cognitive impairment that affected the patient’s ability to complete the questionnaires; (5) patients with worker’s compensation issues; and (6) inadequate follow-up (i.e., less than 24 weeks post-operation).

**Fig. 1:**
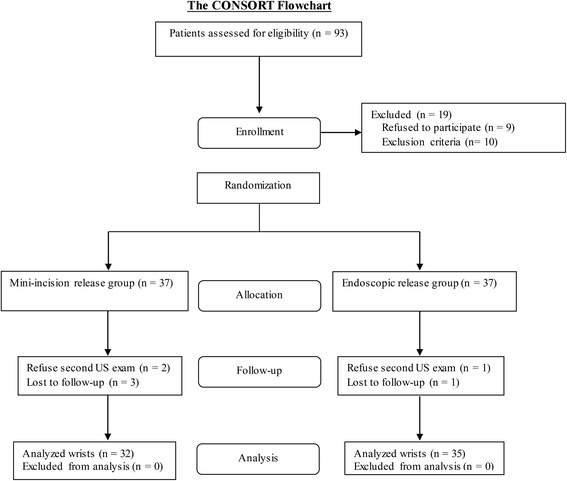
The randomization scheme for the study as demonstrated in the CONSORT flowchart

Based on these criteria, four patients with a history of a distal radius fracture, two patients who required a revision carpal tunnel release, eight patients with at least one of the associated diseases mentioned above, and three patients with worker’s compensation issues were excluded from the study. Another nine patients refused to participate in the study. Patients who met the inclusion criteria and agreed to participate in the study were randomly assigned to receive either the mini-incision or endoscopic release. Block randomization was performed with a computer-generated table of random numbers (Fig. 1) to ensure an equal distribution between groups. Neither the patients nor the treating physicians were blinded to the treatment group due to the unique incision scar inherent to each technique.

Initially, 37 patients were enrolled in each group; however, two patients in the mini-incision release group and one patient in the endoscopic release group refused to undergo another US examination. Three patients in the mini-incision release group and one patient in the endoscopic release group were lost to follow-up. Therefore, 32 patients in the mini-incision release group and 35 patients in the endoscopic release group were included in the final analysis. Demographic information, including sex, age, occupation, duration of symptoms, pre-operative electrophysiologic grade, and disability scores, was collected in all patients. The occupation of each patient was classified into one of three categories: “office worker”, “manual laborer”, or “homemaker” [22]. The electrophysiologic grade was determined on the basis of the American Association of Electrodiagnostic Medicine criteria for electrophysiological test findings [25]. All but one patient in the mini-incision release group and two in the endoscopic release group were right-handed. Twenty-eight patients in the mini-incision release group and twenty-five patients in the endoscopic release group underwent bilateral carpal tunnel release for recalcitrant bilateral CTS; however, only the results from the dominant extremity were included in the analysis. The sex, age, occupation, duration of symptoms, pre-operative electrophysiologic grade, and disability scores were similar across the mini-incision and endoscopic groups (Tables 1 and 2).

**Table 1:** Demographics and Baseline Data

Variables	Mini-incision release group (*n* = 32)	Endoscopic release group (*n* = 35)	*P*-value*
Age (yr)	51.8 ± 10.4	52.9 ± 10.6	0.694
Male/Female (n)	5/27	5/30	0.878
Occupation (n (%))			0.867
Office worker	4 (13)	6 (17)	
Manual worker	2 (6)	2 (6)	
Homemaker	26 (81)	29 (77)	
Bilateral wrists affected (n (%))	28 (88)	25 (71)	0.109
Duration of symptoms (mo)	41.3 ± 57.5	44.3 ± 58.1	0.815
Electrodiagnostic grade (n (%))			0.956
Mild	3 (9)	4 (11)	
Moderate	21 (66)	24 (69)	
Severe	8 (25)	9 (20)	
BCTQ score			
Symptom score	3.2 ± 0.9	3.1 ± 0.8	0.734
Functional score	2.8 ± 1.1	2.7 ± 1.0	0.827
DASH score	46.0 ± 21.6	45.5 ± 19.9	0.921
CSA-I (mm^2^)	13.2 ± 4.6	13.0 ± 6.0	0.885
CSA-M (mm^2^)	8.4 ± 3.2	8.9 ± 4.0	0.543
CSA-O (mm^2^)	7.0 ± 2.3	7.3 ± 2.8	0.657
FR-M	3.6 ± 0.8	3.8 ± 0.9	0.550
FR-O	4.3 ± 0.9	4.3 ± 0.8	0.816

Continuous values are mean ± standard deviation. *BCTQ* Boston Carpal Tunnel Questionnaire, *DASH* Disabilities of Arm, Shoulder and Hand, *CSA-I* Cross-Sectional Area - Inlet, *CSA-M* Cross-Sectional Area - Middle, *CSA-O* Cross-Sectional Area - Outlet, *FR-M* Flattening Ratio - Middle, *FR-O* Flattening Ratio – Outlet. *P values are calculated using the Pearson’s chi-squared test and the Cochran-Armitage trend test for categorical variables, and a two sample *t*-test and the Wilcoxon rank sum test for continuous variables

**Table 2:** Clinical and Ultrasonographic Outcomes at 24 Weeks Post-operation

Variables	Mini-incision release group	Endoscopic release group	*P*-value
BCTQ score			
Symptom score	1.3 ± 0.3	1.2 ± 0.2	0.155
Function score	1.2 ± 0.2	1.2 ± 0.2	0.549
DASH score	8.7 ± 4.7	7.5 ± 4.5	0.292
CSA-I (mm^2^)	9.9 ± 2.5	10.1 ± 2.8	0.778
CSA-M (mm^2^)	11.4 ± 2.6	12.0 ± 2.8	0.404
CSA-O (mm^2^)	10.8 ± 2.4	10.0 ± 1.6	0.114
FR-M	3.2 ± 0.7	3.2 ± 0.6	0.938
FR-O	3.0 ± 0.6	2.9 ± 0.7	0.682

The values are mean ± standard deviation. *BCTQ* Boston Carpal Tunnel Questionnaire, *DASH* Disabilities of Arm, Shoulder and Hand, *CSA-I* Cross-Sectional Area - Inlet, *CSA-M* Cross-Sectional Area - Middle, *CSA-O* Cross-Sectional Area - Outlet, *FR-M* Flattening Ratio - Middle, *FR-O* Flattening Ratio – Outlet. *P values are calculated using a two sample *t*-test and the Wilcoxon rank sum test

The carpal tunnel release procedures were performed by one senior hand surgeon (YC) using regional or general anesthesia. The limb was exsanguinated with an elastic bandage and a pneumatic tourniquet was inflated. The arm distal to the tourniquet was exposed. In the mini-incision release group, a 1.5-cm incision was made distally in the proximal palm over the transverse carpal ligament, beginning from the intersection of Kaplan’s cardinal line, which is drawn between the radial aspect of the thumb web space in abduction and the hook of hamate, with a line drawn along the radial border of the ring finger. After the skin incision, the subcutaneous tissue was incised with a no. 15 blade and retracted laterally. The distal portion of the transverse carpal ligament was divided. A subcutaneous tunnel was made over the transverse carpal ligament using a curved mosquito hemostat, and a standard nasal speculum was introduced into the subcutaneous tunnel. First the proximal portion and then the distal portion of the transverse carpal ligament was released under direct vision. In the endoscopic release group, the Agee technique, which was previously described by Ruch and Poehling [23], was used. A 1.5-cm sized transverse incision was made in the proximal wrist crease between the tendons of the palmaris longus and flexor carpi ulnaris. The forearm fascia was exposed with a blunt dissection, and incised and elevated toward the palmar aspect to create an opening at the carpal tunnel. The tunneling tools were used to dilate the opening, and the synovium from the deep surface of the transverse carpal ligament was scraped. The blade assembly was inserted into the carpal tunnel with the wrist slightly extended. The blade assembly was kept in alignment with the ring finger and hugged the hamate hook by staying to the ulnar side. The distal edge of the transverse carpal ligament was defined by the layer of overlying fat. After the blade assembly was correctly aligned, the blade was elevated and withdrawn to incise the ligament.

Following the complete release of the transverse carpal ligament with each technique, the tourniquet was removed and the wound was closed with 4–0 nylon sutures. A soft bulky dressing was placed, but no splint was applied. The patients were encouraged to move their hands immediately after surgery.

An independent observer who was blinded to the surgical technique performed pre-operative and post-operative assessments of symptom severity and function using both the Boston Carpal Tunnel Questionnaire (BCTQ) [18] and the Disabilities of the Arm, Shoulder, and Hand (DASH) questionnaire [10]. The BCTQ is a disease-specific status scale that incorporates both a symptom severity scale and a functional scale. The symptom severity scale (BCTQ-S) is comprised of eleven items that address the severity, frequency, and duration of symptoms, whereas the functional status scale (BCTQ-F) is comprised of eight questions that assess the difficulty of performing eight daily tasks. Each question offers five possible responses of increasing severity, which are scored from 1 (none) to 5 (most severe); the mean values of all the items in the BCTQ were calculated. The DASH quantifies general disabilities related to the upper extremity. The questionnaire contains 30 items: 21 questions that assess difficulties with specific tasks, five that evaluate symptoms, and four that evaluate social function, work function, sleep, and confidence. The DASH scores are scaled between 0 and 100 with higher scores representing greater upper extremity disability.

**Fig. 2:**
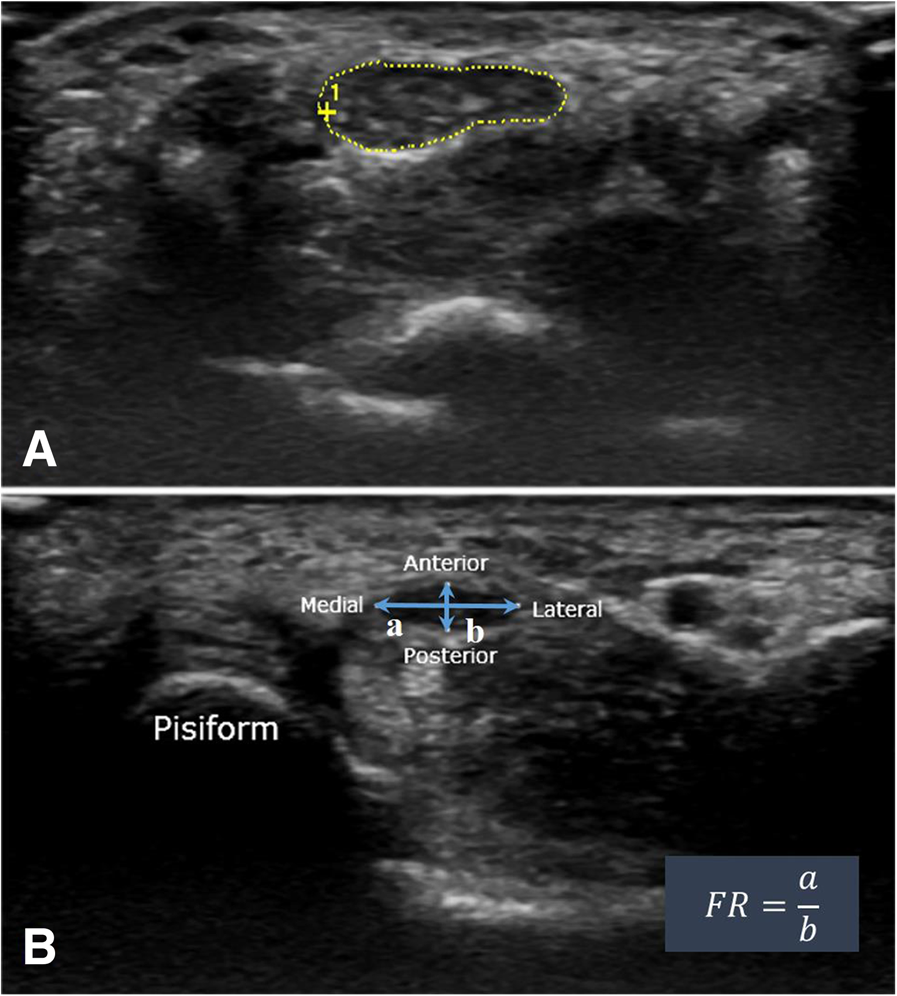
**a** A transverse US scan to measure the cross-sectional area (CSA) of the median nerve at the tunnel inlet, which was at the level of the distal wrist crease. The CSAs of the nerve at the middle of the tunnel at the level of the pisiform and the tunnel outlet at the level of the hamate hook were also measured. **b** The flattening ratio (FR) of the median nerve, calculated by dividing the transverse diameter of the nerve by the anteroposterior diameter

Each patient underwent an ultrasound (US) examination by a radiologist pre-operatively and 24 weeks post-operation using a scanner with a 12/5-MHz linear array transducer (GE Healthcare LOGIQ S6, Milwaukee, WI). During the examination, the patient sat in a comfortable position facing the examiner. The measured forearm rested on the table with the arm supinated, the wrist in a neutral position, and the fingers semi-extended (naturally extended to about half of full extension) [16]. The transducer was placed directly on the patient's skin with gel. The median nerve was first imaged in a longitudinal scan with the US transducer placed at the midline between the radius and ulna and the center of the transducer placed at the distal wrist crease to obtain an initial general overview of the median nerve. This overview of the median nerve was then used to help the examiner obtain optimal axial images. A transverse scan was performed to record the CSA by keeping the transducer directly perpendicular to the long axis of the median nerve. The CSA was calculated by a continual tracing of the nerve circumference excluding the hyper-echoic epineurial rim [3]. Measurements were conducted at the tunnel inlet at the distal wrist crease level (CSA-I), the middle of the tunnel at the level of the pisiform (CSA-M), and the tunnel outlet at the level of the hamate hook (CSA-O) (Fig. 2a). The flattening ratio (FR) of the median nerve was also calculated by dividing the transverse diameter of the nerve by the anteroposterior (AP) diameter of the nerve at the middle (FR-M) and outlet (FR-O) of the carpal tunnel (Fig. 2b). The radiologist performed assessments twice at each time point and recorded the arithmetic mean of the two assessments.

SPSS Statistics version 19.0 (SPSS, Inc., IBM®, Chicago, IL, USA) was used for all of the statistical analyses. A difference of 10 points between the DASH scores of the two groups was considered to be the minimal clinically important difference [1]. Based on our previous study [11], a sample size calculation determined that we would have 90% power to observe a clinically significant difference at an alpha of 0.05 with 32 cases per group.

We aimed to enroll 74 patients to account for an anticipated drop-out rate of approximately 15%. Group results were compared using either the Pearson’s chi square or Fisher’s exact test for the categorical variables and the paired *t*-test and two sample *t*-test for the continuous variables. Correlation analyses were performed to identify relationships between US-based morphological changes and improvements in the subjective outcomes (i.e., BCTQ and DASH scores) following mini-incision or endoscopic carpal tunnel release surgery 24 weeks post-operation. For all analyses, the level of significance was set at *p* < 0.05.

## Results

The BCTQ and DASH scores improved in both groups 24 weeks post-operation compared with the pre-operative scores. The mean (and standard deviation) BCTQ-S score improved from 3.2 ± 0.9 pre-operation to 1.3 ± 0.3 post-operation in the mini-incision release group and 3.1 ± 0.8 pre-operation to 1.2 ± 0.2 post-operation in the endoscopic release group. The mean BCTQ-F score improved from 2.8 ± 1.1 pre-operation to 1.2 ± 0.2 post-operation in the mini-incision release group and 2.7 ± 1.0 pre-operation to 1.2 ± 0.2 post-operation in the endoscopic release group. The mean DASH score also improved from 46.0 ± 21.6 pre-operation to 8.7 ± 4.7 post-operation in the mini-incision release group and 45.5 ± 19.9 pre-operation to 7.5 ± 4.5 post-operation in the endoscopic release group. There were no major differences in the subjective outcomes 24 weeks post-operation compared with the pre-operation scores between the mini-incision and endoscopic release groups (Table 2). There were also no serious operation-related complications, such as deep wound infections, median nerve injury, or the need for a revision carpal tunnel release.

In the mini-incision release group, the mean (and standard deviation) CSA-I was decreased from 13.2 ± 4.6 mm^2^ to 9.9 ± 2.5 mm^2^ (*p* < 0.001). In contrast, the mean CSA-M and CSA-O were increased from 8.4 ± 3.2 and 7.0 ± 2.3 mm^2^ to 11.4 ± 2.6 and 10.8 ± 2.4 mm^2^, respectively (*p* < 0.001 and *p* < 0.001, respectively). In the endoscopic release group, the mean ± SD CSA-I was decreased from 13.0 ± 6.0 mm^2^ to 10.1 ± 2.8 mm^2^ (*p* < 0.001). In contrast, the mean CSA-M and CSA-O were increased from 8.9 ± 4.0 and 7.3 ± 2.8 mm2 to 12.0 ± 2.8 and 10.0 ± 1.6 mm^2^, respectively (*p* < 0.001 and *p* < 0.001, respectively). In the mini-incision release group, the mean FR-M/O decreased from 3.6 ± 0.8/4.3 ± 0.9 to 3.2 ± 0.7/3.0 ± 0.6 (*p* = 0.021 and *p* < 0.001, respectively). In the endoscopic release group, the mean FR-M/O decreased from 3.8 ± 0.9/4.3 ± 0.8 to 3.2 ± 0.6/2.9 ± 0.7 (*p* < 0.001 and *p* = 0.018, respectively). There were no significant differences in the changes in the mean CSA at each level and the mean FR at the middle and outlet between the two groups 24 weeks post-operation (Table 2).

**Fig. 3:**
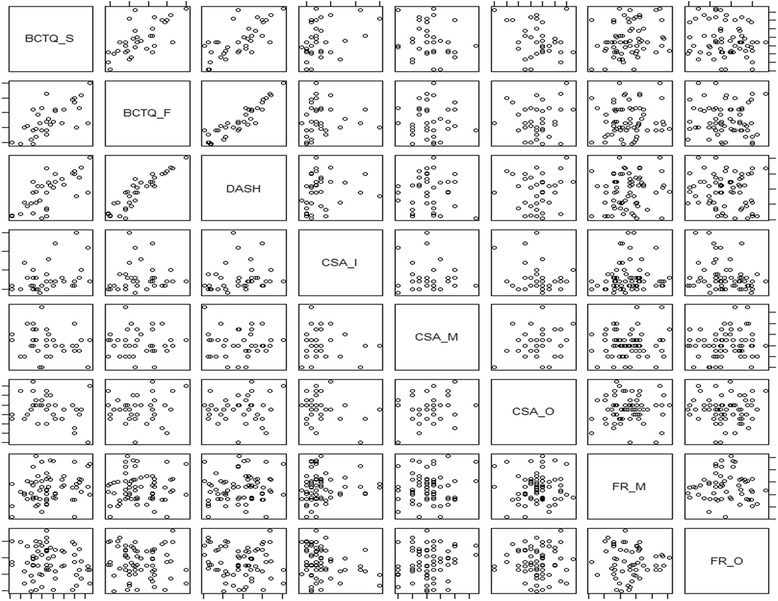
Pairwise scatter plot of difference values for the clinical outcomes and the sonographic outcomes. Positive values indicate improvement of outcomes. Improvement in BCTQ-S score was significantly correlated with improvement in CSA-I (*p* = 0.005), while other values were not. BCTQ = Boston Carpal Tunnel Questionnaire; DASH = Disabilities of Arm, Shoulder and Hand; CSA-I = Cross-Sectional Area - Inlet; CSA-M = Cross-Sectional Area - Middle; CSA-O = Cross-Sectional Area - Outlet; FR-M = Flattening Ratio - Middle; FR-O = Flattening Ratio – Outlet

There were no correlations between the improvements in the mean BCTQ-F or DASH score and the US changes in CSA at each level of carpal tunnel and FR in the middle or distal carpal tunnel 24 weeks post-operation. The improvement in the mean BCTQ-S score was significantly correlated with the decrease in CSA-I (*p* = 0.005). The correlation coefficient of BCTQ-S with CSA-I was 0.38 (95% confidence interval, 0.15–0.57) (Fig. 3).

## Discussion

The mini-incision and endoscopic release of the transverse carpal ligaments are the most common procedures for patients with recalcitrant CTS. Although each technique has advantages and disadvantages, the subjective outcomes 3 months or more after surgery are similar [11, 22, 27]. A few studies have shown that the CSA at the carpal inlet is decreased after either mini-incision or endoscopic carpal tunnel release [17, 24, 26]. However, no studies have examined whether the mini-incision and endoscopic carpal tunnel release techniques have an equal effect on the morphological changes in the median nerve or whether those changes correlate with improvements in assessments of symptom severity and disability.

There were several limitations in this study. First, we included patients who underwent a bilateral release for CTS. Although we analyzed the results from the dominant extremity only, the DASH scores of these patients may have been affected by the non-dominant extremity. However, there was no significant difference in the number of bilateral releases performed in in the mini-incision compared with the endoscopic release group (28 vs. 25 patients, respectively; *p* = 0.109). Second, neither the patients nor the treating physicians were blinded to the surgical technique due to the unique incision scars inherent to each technique. An independent observer (BRK), who was not directly involved in the care of the patients, evaluated the subjective pain and disability of each patient during the post-operation follow-up. Finally, the CSA was evaluated and compared as an absolute value, not a proportional value or difference. As CSA was generally lager in males relative to females, this could be a confounding factor. However, there was no significant difference in the sex ratio between two groups.

Our first aim was to investigate whether subjective outcomes would differ 24 weeks post-operation between the mini-incision and endoscopic release groups. In this study, our overall clinical outcome is consistent with the clinical outcomes that have been observed in previous studies of mini-incision or endoscopic release [1, 6, 9, 19, 25, 27]. In addition, no significant differences were observed in the subjective outcome measures between the mini-incision and endoscopic release groups (*p* > 0.05). Release of the transverse carpal ligament by either method relieved symptoms and improved function by decompressing the carpal tunnel. This decompression also increased the volume and CSA of the median nerve [15]. From these results, we hypothesize that a transection of the transverse carpal ligament induces morphological changes in the median nerve.

An increase in the CSA of the median nerve at the inlet of the carpal tunnel and a flattening of the median nerve in the distal carpal tunnel are distinctive features of CTS [7, 12, 13, 17, 20, 21]. A few studies have shown a decrease in the pathologic swelling of the median nerve at the inlet of the carpal tunnel and an increase in the flattening ratio of the median nerve in the carpal tunnel with mini-incision release [17, 24, 26]. Likewise, several publications on endoscopic release have also reported decreased CSA and increased FR of the median nerve in US measurement post-operatively [2, 5]. However, few studies have compared pathological changes for an open technique to an endoscopic technique. We therefore conducted repeated US examinations of the median nerve at the inlet, outlet, and middle of the carpal tunnel pre-operatively and 24 weeks post-operation by the same radiologist, and comparing the outcomes of both surgical groups. In both groups, the CSA of the median nerve was significantly decreased at the inlet of the carpal tunnel. In contrast, the CSA of the median nerve was significantly increased at the middle and outlet of the carpal tunnel. The change in the area at each level of the carpal tunnel from the pre-operative to the post-operative measure was similar in both groups. In addition, the flattening ratio of the median nerve significantly decreased in both groups post-operation compared with the pre-operative measure. This similarity in morphological changes following surgery may explain why both the mini-incision and endoscopic carpal tunnel release groups reported similar subjective outcomes.

We also analyzed the correlations between the improvements in the BCTQ and DASH scores with the morphological changes in the median nerve. The improvement in the BCTQ-S score was significantly correlated with a decrease in the CSA at the inlet of the carpal tunnel (*p* = 0.005). These results suggest that improvements in symptoms, but not function, are associated with a decrease in median nerve swelling at the inlet of the carpal tunnel.

## Conclusions

Both mini-incision and endoscopic carpal tunnel release significantly reversed the pathological changes in the median nerve morphology of patients with CTS, with no significant differences between techniques. Symptom relief after surgical decompression seems to correlates with the decreased nerve swelling at carpal inlet. This study suggests that the similar subjective outcomes following mini-incision and endoscopic release can result from similar morphologic changes in the median nerve following surgical decompression.

## Abbreviations

CTS: Carpal tunnel syndrome
BCTQ: Boston carpal tunnel questionnaire
DASH: Disabilities of the arm, shoulder, and hand, disabilities of the arm, shoulder, and hand
CSA-I: CSA-M and CSA-O, Cross-sectional area the inlet, middle, and outlet
FR-M and FR-O: Flattening ratio at the middle and outlet
US: Ultrasonography
